# Cost and Length of Hospitalization Associated with Dental Infections: A Systematic Review

**DOI:** 10.3390/ijerph23020259

**Published:** 2026-02-18

**Authors:** Somayeh Parvizi, Albert Yaacoub, Stephen Cox, Carlos Marcelo da Silva Figueredo, Mafaz Ullah

**Affiliations:** 1 Discipline of Oral Surgery, Faculty of Medicine and Health, University of Sydney, Sydney, NSW 2750, Australia; somayeh.parvizi@sydney.edu.au (S.P.); albert.yaacoub@health.nsw.gov.au (A.Y.); stephen.cox@sydney.edu.au (S.C.); 2 Nepean Centre for Oral Health, Nepean Blue Mountains Local Health District, Kingswood, Sydney, NSW 2747, Australia; 3 School of Nursing and Midwifery, Western Sydney University, Locked Bag 1797, Penrith, Sydney, NSW 2751, Australia; 4 School of Medicine and Dentistry, Griffith University, Gold Coast, QLD 4215, Australia; c.dasilvafigueredo@griffith.edu.au; 5 Department of Dental Medicine, Division of Oral Diseases, Karolinska Institute, SE-171 77 Stockholm, Sweden

**Keywords:** dental infection, hospitalization, cost, economic burden, systematic review

## Abstract

**Highlights:**

**Public health relevance—How does this work relate to a public health issue?**

- Dental infections are a major cause of potentially preventable hospital admissions, contributing substantially to healthcare utilization and costs worldwide.
- This systematic review synthesizes global evidence on hospital length of stay and financial burden associated with dental infection–related hospitalizations.

**Public health significance—Why is this work of significance to public health?**

- The review demonstrates wide variability in hospital length of stay (1.15–10 days) and costs (AUD 2,402 to USD 47,835.60), highlighting a significant and growing strain on healthcare systems.
- Findings emphasize dental infections as an under-recognized public health issue with serious economic and clinical consequences.

**Public health implications—What are the key implications or messages for practitioners, policy makers and/or researchers in public health?**

- Strengthening preventive dental care, early intervention, and timely access to treatment could substantially reduce avoidable hospitalizations and healthcare expenditure.
- Policymakers and healthcare planners should prioritize oral health within public health strategies to mitigate the clinical and economic burden of severe dental infections.

**Abstract:**

Objectives: This study aimed to investigate the cost of hospitalization and length of stay (LOS) associated with dental infections and their impact on healthcare resources. Materials and Methods: Web of Science, Medline via Ovid, and Google Scholar were searched for articles published from 2013 to 2023 using relevant MeSH terms. A descriptive summary was produced to describe study characteristics, and selected studies were analyzed to evaluate financial implications and hospitalization outcomes. Results: After removing duplicates, 125 articles were screened, with 25 read in full and 9 excluded. Sixteen retrospective studies were included, encompassing 156,487 patients. Hospital length of stay ranged from 1.15 to 10 days, and hospitalization costs ranged from AUD 2402 to USD 47,836.60. Variability in outcomes appeared to be influenced by infection severity, healthcare infrastructure, and geographic setting. Conclusions: Dental infections impose a substantial and variable burden on healthcare systems. These findings emphasize the need for timely intervention, preventive strategies, and standardized management protocols to reduce clinical complications and financial strain on healthcare services.

## 1. Introduction

Dental infections, also known as odontogenic infections, arise from bacteria in the tooth or supporting structures [1]. Their origin and progression are complex, involving both local and systemic factors. Local etiological factors include dental caries, pericoronitis, periodontal diseases, dental trauma, defective restorations, and failed root canal treatments [2]. Systemic factors, such as medical conditions or medications that suppress immune responses or reduce tissue perfusion and salivary flow, including radiotherapy, chemotherapy, diabetes, and Sjögren’s syndrome, also influence infection progression [3]. The interplay of these factors, along with genetic, environmental, and cultural influences and oral hygiene practices, contributes to the development and severity of dental infections [4,5].

Dental caries, the primary precursor of dental infections, is initiated by demineralization caused primarily by *Streptococcus mutans* and *Lactobacillus* species. If untreated, infection may extend from the dentin to the pulp and beyond the apical region, potentially spreading into facial spaces and causing cellulitis or abscesses. Severe cases can result in life-threatening complications, such as Ludwig’s angina or cavernous sinus thrombosis [6,7].

Dental infections leading to facial cellulitis and abscess formation often require hospital management and are significant contributors to potentially preventable hospitalizations [8]. Hospitalization places a substantial burden on healthcare systems, including costs from emergency visits, hospital stays, ICU admissions, surgical interventions, and theater resources [9,10]. In the United States, the average cost per patient for acute dental infection management was USD 28,841 [11], while in South Australia it was AUD 12,228 [12]. National dental expenditure in Australia in 2020–2021 was AUD 11.1 billion, of which only AUD 1.2 billion was government-funded [12]. In the United States, annual inpatient costs for dental infections were estimated at approximately USD 200 million [13].

This study reviews the literature on hospital length of stay (LOS) and hospitalization costs associated with dental infections, aiming to evaluate their impact on healthcare resources.

## 2. Materials and Methods

### 2.1. Review Question

This systematic review aimed to answer the research question:

“What are the costs of hospitalization and length of hospital stay associated with dental infections?”

By reviewing published literature from 2013 to 2023, the study sought to provide insights into the financial burden of dental infections requiring hospital care and their potential impact on healthcare resources and expenditures.

### 2.2. Search Strategy

Database selection: A comprehensive literature search was conducted using Web of Science, Medline via Ovid, and Google Scholar (limited to the first 200 records ranked by relevance) for studies published between 2013 and 2023.

Search terms: Appropriate MeSH terms were identified based on the research question, the MeSH hierarchy, and relevance to dental infections and hospital resource use. Boolean operators (AND, OR) were applied to combine terms into a comprehensive search strategy. The following terms were included:

- Ludwig’s Angina OR Periapical Abscess OR Periapical Periodontitis OR dentoalveolar infection OR dentoalveolar abscess
- Dental Caries OR tooth decay OR periapical infection OR Pericoronitis OR odontogenic cellulitis
- Hospitalization OR hospital stays OR hospital admission OR Length of Stay
- Costs OR Cost Analysis

**Figure 1:**
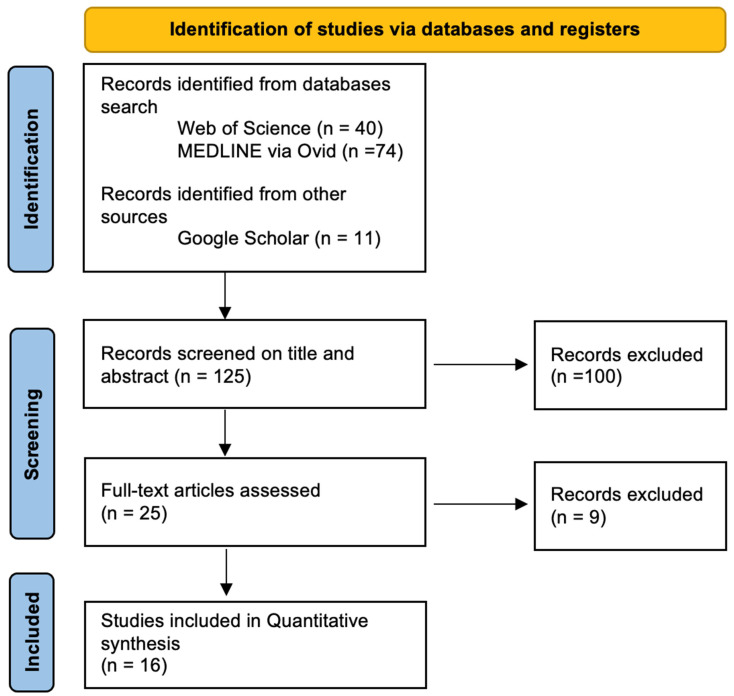
The flow chart demonstrates the sequential steps involved in selecting studies for this review.

Search results were exported to EndNote, and duplicates were removed. Titles and abstracts were screened independently by two reviewers (S.P. and M.U.) to assess relevance. Full texts of potentially eligible studies were then retrieved and assessed. The study selection process is summarized in a flowchart (Figure 1).

### 2.3. PRISMA Compliance

This review was conducted and reported following the PRISMA 2020 guidelines. A completed PRISMA checklist and flow diagram illustrating the study selection process are included in the manuscript (Figure 1) and provided as Supplementary Materials. This systematic review was not prospectively registered in PROSPERO. While prospective registration is recommended, it was not conducted due to the retrospective nature of the review and the limited time prior to study initiation. All methods, including search strategy and eligibility criteria, were predefined to maintain methodological transparency.

### 2.4. Inclusion and Exclusion Criteria

- Inclusion criteria: Studies published in English between 2013 and 2023, reporting on hospitalization costs and/or length of hospital stay due to dental infections. All included studies were retrospective observational analyses.
- Exclusion criteria: Studies on non-odontogenic infections or those not reporting costs or hospital stay related to dental infections. No prospective or interventional studies meeting the inclusion criteria were identified.

### 2.5. Quality Evaluation

The methodological quality of the included economic studies was assessed using a modified version of the Drummond and Jefferson criteria [14] (Table 1). Each quality criterion was categorized as positive, negative, or unclear. Studies that received positive ratings for 50% or more of the criteria were classified as having strong methodological rigor, while those with less than 50% positive ratings were considered to have lower methodological quality. The 50% threshold was chosen arbitrarily to create two distinct categories, distinguishing studies with generally adequate methodological rigor from those with potential limitations.

Across the 16 included studies, the average quality score was 6.5 out of 10. The criteria for assessment were based on the Drummond and Jefferson framework [14].

The quality of each study was evaluated based on the following modified Drummond and Jefferson criteria [14], with each item rated as Yes (+), No (−), or Cannot tell (N/A):

1. Was a well-defined question asked in an answerable form?
2. Was a comprehensive description of the competing alternatives provided?
3. Was there evidence that the program’s effectiveness was established?
4. Were all important and relevant costs and consequences identified?
5. Were costs and consequences measured accurately using appropriate physical units?
6. Were costs and consequences credibly valued?
7. Were costs and consequences adjusted for differential timing?
8. Was an incremental analysis of costs and consequences of alternatives performed?
9. Was a sensitivity analysis performed?
10. Did the presentation and discussion of the study results address all issues of concern to users?

Although this modified Drummond and Jefferson framework was applied to assess methodological quality, no formal risk-of-bias assessment tool was used. This is acknowledged as a methodological limitation of the review.

### 2.6. Summary

- Strong methodological quality: 10/16 studies
- Low methodological quality: 6/16 studies
- Average score across all studies: 6.5/10

## 3. Results

Search Outcomes: The initial search identified 125 articles. After screening titles and abstracts, 25 full-text articles were assessed for eligibility, of which 16 retrospective studies (Table 2) met the inclusion criteria for detailed review.

Figure 1 presents the PRISMA flow diagram, illustrating the study selection process along with the corresponding numbers of identified and included articles. All included studies employed a retrospective design, with considerable variation in study duration and sample sizes.

The 16 included studies originated from multiple countries: six from the United States (USA) [15,16,17,22,26,29], four from Australia [12,20,23,27], and one each from Brazil [18], Germany [24], Poland [28], Lithuania [21], Turkey [19], and the United Kingdom (UK) [25]. Collectively, these studies encompassed a total of 156,487 patients hospitalized due to dental infections (Table 2).

### 3.1. Demographic Results

The mean age of hospitalization ranged from 36 to 43 years across studies that included all age groups (Table 3). In the study by Kruger & Tennant [20], approximately 44% of admissions involved children under 14 years old. Conversely, Kara et al. [19] and the most recent study by Doll et al. [24] included patients under 18 years old exclusively, with mean ages of 7.28 ± 3.26 years and 6.3 years, respectively. In Gonçalves et al. [18], which included patients under 60 years old, the 0–10-year age group was the most prevalent, followed by the 31–40-year age group.

Regarding gender (Table 3), three studies with larger sample sizes reported a slightly higher proportion of females: 51.1% [17], 52% [20], and 53% [26], whereas in the other 11 studies with sample sizes under 500, males were slightly more represented. In Gonçalves et al. [18] and Liau et al. [23], no significant gender differences were observed.

### 3.2. Length of Stay (LOS) and Cost of Hospitalization

The primary outcomes extracted from included studies were hospitalization costs and length of hospital stay (LOS) associated with dental infections. Length of hospital stay (LOS) was reported variably across studies, including mean, median, ranges, and in some cases, separate ICU and ward stays. For consistency, we report LOS as presented in the original studies and note when ICU stay is included separately. Hospitalization costs were reported in different currencies and years; these were retained as originally published. For clarity and potential comparison, all costs could be converted to a single reference currency and year using purchasing power parity (PPP) and inflation adjustments, though this was not performed in this review to preserve the original reported values. Cost data were analyzed descriptively rather than pooled or standardized due to substantial differences in healthcare systems, reimbursement models, currencies, and study periods across countries. The reported hospitalization costs in 12 studies ranged from AUD 2,402 [20] to USD 47,835.60 [29] (Table 3) and were reported in GBP, AUD, EUR, and USD.

These costs may not be directly comparable to current values due to the time gap between data collection and publication, as well as variations in currency values across countries. This variability likely influences the reported ranges of hospital LOS and costs, limiting direct comparability across studies. Future research could consider relative metrics, such as cost as a percentage of GDP per capita or average annual salary, to enable more standardized cross-country comparisons.

The shortest mean hospital stay was reported by Kruger & Tennant [20] at 1.15 days, whereas Rūta Rastenienė et al. [21] reported the longest mean stay at 8.3 ± 4.9 days. Over an 8-year period, hospital costs in one study exceeded USD 3.3 million, with submitted charges surpassing USD 10 million [15]. Similarly, Christensen et al. [16] reported a total hospital bill of USD 5,422,854 over 10 years.

Shah et al. [17] reported total hospital charges, adjusted for inflation, of AUD 858.9 million. According to Kruger & Tennant [20], total costs for avoidable hospitalizations related to oral health were estimated at approximately AUD 157 million, with average costs ranging from AUD 1,655 to 3,150 per patient in 2008–2009 (Table 3). Gams et al. [22] investigated severe odontogenic infections, reporting that 45% of patients required ICU admission, with an average cost per patient of AUD 13,058 and a hospital bill of AUD 48,351 per patient.

In a study of 285 patients with severe odontogenic infections, Rūta Rastenienė et al. [21] found no statistically significant difference in the length of hospital stay (LOH) between patients who sought prompt professional care (8.7 ± 4.7 days) and those who delayed care at outpatient facilities (9.0 ± 8.8 days). Although the delayed-care group exhibited greater variability in hospital stay, this difference was not statistically significant. The overall mean LOS for the study population was 8.3 ± 4.9 days.

Liau et al. [23], studying severe odontogenic infections admitted to the Australian, Royal Adelaide Hospital over 9 years, reported a mean LOS of 4.18 days and a mean ICU stay of 39.12 days. Risk stratification followed the institutional Acute Head and Neck Infection protocol, categorizing patients into high-risk and low-risk groups. This system prioritizes patients at risk of airway compromise for prompt, aggressive treatment. High-risk infections are characterized by dyspnea (especially when supine), dysphagia, odynophagia, lateral laryngeal displacement, trismus (<2 cm mouth opening), or significant intraoral swelling, while low-risk infections involve localized infection without airway compromise indicators [23,24].

Nadig and Taylor [25] reported a total expenditure of GBP 95,200 for 100 patients, highlighting the high cost of largely preventable conditions. In Australia, Han et al. [12] documented hospital expenses associated with dental conditions of AUD 5,649,249, adjusted for inflation, over a seven-year period (2006–2014). Fu et al. [27] estimated ICU costs of USD 765,000 for 170 days of intensive care for advanced odontogenic infections. Neal et al. [29] reported substantial cost differences between dental emergency protocols, with Group A (OISS ≥ 5) incurring USD 4,128,529.78 and Group B (OISS < 5) incurring USD 2,759,796, highlighting the need for preventive, cost-effective dental care strategies. The reported ranges of LOS and hospitalization costs reflect heterogeneity in study populations, infection severity, and healthcare settings. Cost data across countries are not directly comparable due to differences in currencies, healthcare systems, and inflation adjustments.

## 4. Discussion

Despite advances in dental and medical care, including advanced diagnostic imaging and widespread antibiotic use, the reviewed literature demonstrates a rising trend in the incidence, severity, and cost of odontogenic infections requiring hospitalization [5]. Even in countries with advanced healthcare systems, 70 deaths were reported due to odontogenic infections, predominantly associated with airway compromise and comorbidities, particularly among patients with higher Charlson Comorbidity Index scores [12]. Implementation of a risk-stratification protocol at the Adelaide Dental Hospital has since prevented further fatalities from airway compromise, following six deaths in earlier years [9].

Untreated dental infections remain potentially fatal. In Taiwan, 1 in 150 hospital admissions for oral and maxillofacial infections resulted in death, mainly among patients with underlying conditions such as diabetes [30]. A Ghanaian study reported a 5.8% mortality rate for severe odontogenic infections [31], while a systematic review by Pucci et al. (2021) found maternal and fetal mortality rates of 5.8% and 13%, respectively, due to odontogenic infections during pregnancy [32].

Several studies document the growing burden of dental infections on hospital systems. For example, Fu et al. [27] reported a doubling of admissions and costs related to odontogenic infections in Australia over a decade, including ICU admissions and the use of broad-spectrum antibiotics. In the U.S., Shah et al. [17] observed a 41.4% increase in hospital discharges for periapical abscesses, and Morón et al. [26] reported dental emergency admissions in Florida rising from 1808 in 2006 to 3542 in 2016, with associated costs increasing from USD 46.1 million to USD 166.5 million.

The socioeconomic impact extends beyond direct healthcare costs. Lima & Buarque [33] reported absenteeism due to oral health issues ranging from 9% to 27%, with presenteeism due to orofacial pain affecting up to 50% of employees. Children with poor oral health were nearly three times more likely to miss school due to dental pain [34]. In the U.S., hospitalization costs for odontogenic infections ranged from USD 13,590 to 47,835.60 per patient [35].

In Western Australia, potentially preventable hospital admissions for oral conditions exceeded those of any other Australian state, surpassing rates in New Zealand (2.15 per 1000 population). Dental caries remains a leading cause of childhood hospitalization in Australia, highlighting public health challenges such as limited preventive care, insufficient dental insurance, delayed treatment, and poor oral health literacy [36,37,38,39].

Timely intervention and improved access to care can prevent costly and life-threatening complications. Despite the effectiveness of preventive measures, treatment delays often result in advanced infections requiring hospitalization [40]. A significant proportion of patients received antibiotics without addressing the source of infection [19], leading to recurrent and more severe disease. Fu et al. [27] reported that two-thirds of patients had previously been prescribed antibiotics by general practitioners or dentists, indicating reliance on antimicrobials over definitive treatment.

Factors influencing hospital Length of Stay (LOS) include age, comorbidities, infection severity, and timing of intervention. Han et al. [12] reported that 64.1% of hospitalized patients were high-risk due to airway-related space involvement, with most undergoing surgical drainage and tooth extraction under general anesthesia (74.5%). Kara et al. [19] demonstrated that extractions performed within 48 h significantly reduced LOS.

Older patients typically experience longer hospital stays, often due to large-space abscesses and systemic comorbidities [12]. Conditions such as diabetes, obesity, and immunosuppression are associated with increased LOS and costs [41]. Higher odontogenic infection severity scores and ASA scores (≥3) also correlate with elevated healthcare expenses.

Gender differences in health-seeking behavior were noted. A review by Lipsky [42] indicated that men are more likely to neglect oral health, delay care, and present with severe conditions, leading to higher hospitalization rates in 11 of 16 studies [43,44,45,46,47]. However, some larger studies observed slightly higher hospitalization rates among women [17,20,26].

Age-related disparities in dental health remain evident. According to the U.S. National Center for Health Statistics (2011–2012), 26% of individuals aged ≥75 had complete tooth loss, compared to 13% of those aged 65–74 [48].

Finally, overreliance on antibiotics was a common finding. The most frequently prescribed regimen was amoxicillin with metronidazole (82.5%) [12]. Some clinicians delayed surgical drainage due to concerns about swelling or local anesthetic efficacy. The NICE 2000 guidelines, which discourage prophylactic removal of asymptomatic third molars, may have inadvertently contributed to infection rates [48]. A growing lack of surgical confidence among general dentists may further hinder early intervention.

Overall, this review highlights the multifactorial burden of odontogenic infections on healthcare systems and underscores the importance of timely, source-focused treatment. Comprehensive, multidisciplinary strategies addressing access, education, and early intervention are essential to reduce both the human and economic toll of these infections.

Moreover, most included studies were conducted in high-income countries with well-resourced healthcare systems, potentially limiting the generalizability of the reported economic burden to low- and middle-income settings. Interpretation of findings related to disease severity is further constrained by heterogeneity in reporting, as severity stratification was inconsistently applied across studies, requiring reliance on narrative synthesis rather than standardized comparative analyses.

## 5. Conclusions

Dental infections impose a significant burden on healthcare systems, with hospital LOS ranging from 1.15 to 10 days and hospitalization costs varying from AUD 2402 to USD 47,835.60 across the included studies. This variability reflects differences in infection severity, healthcare infrastructure, and geographic context [12,20,21,26,29].

These findings highlight the critical need for timely access to dental care, improved public health strategies, and the adoption of standardized assessment tools, such as the Odontogenic Infection Severity Score (OISS). Enhanced awareness among healthcare providers and policymakers is essential to prioritize preventive dental services, optimize management, and mitigate the clinical and economic impact of odontogenic infections.

## Figures and Tables

**Table 1:** Overall methodological quality of included studies.

Author	Total Score (Out of 10)	Overall Quality
Ahmed et al., [15]	7	Strong
Christensen et al., [16]	6	Strong
Shah et al., [17]	8	Strong
Gonçalves et al., [18]	4	Low
Kara et al., [19]	3	Low
Kruger & Tennant, [20]	7	Strong
Rūta Rastenienė et al., [21]	5	Low
Gams et al., [22]	6	Strong
Liau et al., [23]	7	Strong
Doll et al., [24]	4	Low
Nadig & Taylor, [25]	5	Low
Morón et al., [26]	7	Strong
Han et al., [12]	6	Strong
Fu et al., [27]	6	Strong
Zawiślak & Nowak [28]	4	Low
Neal et al., [29]	6	Strong

**Table 2:** Characteristics of Included Studies.

Author	Country	Year	Study Type	Study Duration (Years)	Data Collection Date	Sample Size (Number)	Mortality (Number)
Ahmed et al., [15]	USA	2013	Retrospective	8	2003 to 2010	327	N/A
Christensen et al., [16]	USA	2013	Retrospective	10	1 July 2001–30 June 2011	318	N/A
Shah et al., [17]	USA	2013	Retrospective	9	2000–2008	1439	66
Gonçalves et al., [18]	Brazil	2013	Retrospective	N/A	N/A	80	N/A
Kara et al., 2014 [19]	Turkey	2014	Retrospective	15	1996–2010	106	N/A
Kruger& Tennant., [20]	Australia	2015	Retrospective	10	1999–2009	65,000	N/A
Rūta Rastenienė et al., [21]	Lithuania	2015	Retrospective	5	2009–2013	85	N/A
Gams et al., [22]	USA	2017	Retrospective	3	January 2010–January 2015	298	N/A
Liau et al., [23]	Australia	2018	Retrospective	9	2006–2014	772	4
Doll et al., [24]	Germany	2018	Retrospective	2	January 2013–March 2015	20	N/A
Nadig & Taylor, 2018) [25]	UK	2018	Retrospective	2	October 2014–September 2016	100	N/A
Morón et al., [26]	USA	2019	Retrospective	11	2006–2016	26,659	N/A
Han et al., [12]	Australia	2019	Retrospective	7	January 2006–December 2014	462	N/A
Fu et al., [27]	Australia	2020	Retrospective	4	January 2003–December 2004 January 2013–December 2014	292	N/A
Zawiślak & Nowak [28]	Poland	2021	Retrospective	1	January 2018–June 2019	85	N/A
Neal et al., [29]	USA	2022	Retrospective	4	January 2016–December 2020	144	N/A

USA: United States; N/A: Not Applicable.

**Table 3:** Data included in the studies.

Author	Age (Years; Range or Eligibility)	Gender Ratio (M:F)	Length of Stay (LOS)	Cost of Admission Per Patient
Ahmed et al., [15]	43 (1–85)	54.7:45.3	N/A	USD 9417
Christensen et al., [16]	39.5 ± 15.9 (≥18)	65.1:34.9	3.5 days	USD 17,053
Shah et al., [17]	37 (range N/A)	48.9:51.1	2.96 days	USD 14,245
Gonçalves et al., [18]	0–10 (35%), 31–40 (22.5%), ≥60	50:50	4.4 days	N/A
Kara et al., [19]	7.28 ± 3.26 (0–17)	58.4:41.6	5.86 ± 3.38 days	N/A
Kruger & Tennant [20]	≤14 (44%), 0–75	48:52	1.15 days	AUD 2402
Rūta Rastenienė et al., [21]	41.5 ± 16.9 (18–90)	58.2:41.8	8.3 ± 4.9 days	N/A
Gams et al., [22]	38.9 (7–77)	55:45	5.5 days	USD 13,058
Liau et al., [23]	N/A	50:50	4.18 days	N/A
Doll et al., [24]	6.3 (≤18)	64:36	1.82 days	EUR 1813.98
Nadig & Taylor [25]	36 (range N/A)	54:46	2.38 days	GBP 1336
Morón et al., [26]	40–49 (17.7%), ≥20	47:53	3.58 days	USD 36,836
Han et al., [12]	39.5 ± 15.9 (≥18)	54:46	4 days	AUD 12,228
Fu et al., [27]	36.14	57.5:42.5	3.73 days	AUD 4500
Zawiślak & Nowak [28]	34.8 ± 14.8 (5–72)	68.2:31.8	5 days (64.7%), 6–10 days (25.9%)	N/A
Neal et al., [29]	41.6 (16–81)	54.8:45.2	5.25 days	USD 47,835.60

AUD = Australian Dollar; USD = United States Dollar; GBP = British Pound; EUR = Euro; N/A = Not Applicable.

## Data Availability

Data supporting the findings of this study are available within the article and its Supplementary Materials.

## Supplementary Materials

The following supporting information can be downloaded at: https://www.mdpi.com/article/10.3390/ijerph23020259/s1, File S1: PRISMA 2020 Checklist [49].
