# Supplementary material for: Cost and Length of Hospitalization Associated with Dental Infections: A Systematic Review
Source: Int J Environ Res Public Health. 2026 Feb 18;23(2):259. doi: 10.3390/ijerph23020259 (PMC12940658; doi:10.3390/ijerph23020259)
Supplement: Supplementary file 1 [file ijerph-23-00259-s001.zip › ijerph-4084550-supplementary.pdf]

PRISMA

PRISMA 2020 Checklist

Section and Topic	Item #	Checklist item	Location where item is reported
**TITLE**			
Title	1	Identify the report as a systematic review.	Title page
**ABSTRACT**			
Abstract	2	See the PRISMA 2020 for Abstracts checklist.	Abstract (structured according to PRISMA 2020 for Abstracts)
**INTRODUCTION**			
Rationale	3	Describe the rationale for the review in the context of existing knowledge.	**Introduction:**<br><br>Paragraph 3 (hospitalisation burden and costs): • It **summarizes existing knowledge** about the burden of dental infections and hospitalisation costs.<br><br>• It **highlights the gap/problem**, i.e., dental infections cause substantial and preventable healthcare costs, which need systematic investigation.<br><br>• It **justifies the review**, which is why this study is needed.
Objectives	4	Provide an explicit statement of the objective(s) or question(s) the review addresses.	Introduction:the **objective (aim)** is the **last paragraph**, which reads:<br><br>*"This study reviews the literature on hospital length………"*
**METHODS**			
Eligibility criteria	5	Specify the inclusion and exclusion criteria for the review and how studies were grouped for the syntheses.	**Covered in methodology**<br><br>• Inclusion: 2013–2023, English, focus on dental infection costs/LOS

PRISMA

PRISMA 2020 Checklist

Section and Topic	Item #	Checklist item	Location where item is reported
			• Exclusion: non-odontogenic infections or not reporting cost/LOS<br>• **grouped for synthesis** (table 1,2 study type/country,sample).
Information sources	6	Specify all databases, registers, websites, organisations, reference lists and other sources searched or consulted to identify studies. Specify the date when each source was last searched or consulted.	Methodology:Selection of online databases: Web of Science, Medline via Ovid, and Google Scholar were utilised to search for relevant literature between 2013 and 2023.
Search strategy	7	Present the full search strategies for all databases, registers and websites, including any filters and limits used.	Methodology part:<br>Medline Via Ovid:<br>1. Ludwig's Angina/ OR Periapical Abscess/ OR Periapical Periodontitis/ OR dentoalveolar infection*.mp. OR dentoalveolar abscess*.mp.<br>2. Dental Caries/ OR tooth decay*.mp. OR periapical infection*.mp. OR Pericoronitis/ OR odontogenic cellulitis*.mp.<br>3. Hospitalization/ OR hospitalisation*.mp. OR "Length of Stay"/ OR hospital stays*.mp. OR hospital admission*.mp.<br>4. Costs and Cost Analysis/<br>5. 1 OR 2<br>6. 3 AND 4 AND 5<br>Limits: English language, published 2013–2023.

Section and Topic	Item #	Checklist item	Location where item is reported
			WOS:<br>TS=("Ludwig's Angina" OR "Periapical Abscess" OR "Periapical Periodontitis" OR "dentoalveolar infection*" OR "dentoalveolar abscess*" OR "Dental Caries" OR "tooth decay*" OR "periapical<br><br>infection*" OR "Pericoronitis" OR "odontogenic cellulitis*")<br>AND TS=("Hospitalization" OR "hospitalisation*" OR "Length of Stay" OR "hospital stays*" OR "hospital admission*")<br>AND TS=("Costs" OR "Cost Analysis")<br>Filters: English, 2013–2023.<br>**Google Scholar (2013–2023, English language)**<br>• States **filters/limits** (English language, 2013–2023)
Selection process	8	Specify the methods used to decide whether a study met the inclusion criteria of the review, including how many reviewers screened each record and each report retrieved, whether they worked independently, and if applicable, details of automation tools used in the process.	After removal of duplicates using **EndNote**, all titles and abstracts retrieved from the searches were **screened independently by two reviewers (S.P. and M.U.)** to assess relevance according to the predefined inclusion and exclusion criteria.

PRISMA

PRISMA 2020 Checklist

Section and Topic	Item #	Checklist item	Location where item is reported
			Articles deemed potentially eligible were then **assessed at full-text level** by the same reviewers. Any discrepancies between reviewers were resolved through discussion, and if needed, a third reviewer would have been consulted to reach consensus. The process of study selection, including numbers of records at each stage, is illustrated in the **PRISMA flow diagram (Figure 1)**.
Data collection process	9	Specify the methods used to collect data from reports, including how many reviewers collected data from each report, whether they worked independently, any processes for obtaining or confirming data from study investigators, and if applicable, details of automation tools used in the process.	Section: Methods section → Data collection / Data items subsection<br>Data were extracted from each included study using a **standardized data extraction form** developed by the reviewers to capture relevant information on study characteristics, hospitalisation costs, length of stay (LOS), infection type, country, study design, and sample size. **Two reviewers (S.P. and M.U.) independently extracted the data** from each report. Discrepancies between reviewers were resolved through discussion, and if consensus was not reached, a third reviewer would be consulted. When data were unclear or missing, attempts were made to contact study authors to obtain or confirm the required information. Extracted data were managed using **Microsoft Excel** to organize and prepare for synthesis.
Data items	10a	List and define all outcomes for which data were sought. Specify whether all results that were compatible with each outcome domain in each study were sought (e.g. for all measures, time points, analyses), and if not, the methods used to decide which results to collect.	**Location in manuscript:** *Methods → Search Strategy / Data Extraction*

Section and Topic	Item #	Checklist item	Location where item is reported
			section (or a separate Data Items subsection if you have one)<br><br>• **Cost of hospitalisation** associated with dental infections (reported in local currency; converted to USD or AUD where applicable for comparison).<br><br>• **Length of hospital stay (LOS)**, measured in days.<br>For each study, all reported results relevant to these outcomes were collected, including means, medians, ranges, and summary statistics.
	10b	List and define all other variables for which data were sought (e.g. participant and intervention characteristics, funding sources). Describe any assumptions made about any missing or unclear information.	**Location in manuscript:** *Materials and Methods → Data collection / Results → Tables 1 & 2*<br><br>• **Study characteristics:** country, year of publication, study design, study duration, and data collection dates (Table 1).<br><br>• **Patient characteristics:** age (mean, range, or age group), gender distribution (male:female ratio) (Table 2), and comorbidities if reported.<br><br>• **Infection characteristics:** type of odontogenic infection (e.g., Ludwig's angina, periapical abscess, dentoalveolar infection, pericoronitis).<br><br>• **Hospital characteristics and interventions:** hospital type/setting, intensive care unit (ICU) admission, surgical interventions, and risk stratification protocols if reported.

PRISMA 2020 Checklist

Section and Topic	Item #	Checklist item	Location where item is reported
			• **Economic and methodological details:** cost per patient, currency, inflation adjustments, total hospitalisation costs, and funding sources where available.
Study risk of bias assessment	11	Specify the methods used to assess risk of bias in the included studies, including details of the tool(s) used, how many reviewers assessed each study and whether they worked independently, and if applicable, details of automation tools used in the process.	**Location in manuscript:** *Materials and Methods → Quality Evaluation*<br><br>The methodological quality and risk of bias of each included study were assessed using a **modified version of the Drummond and Jefferson economic evaluation criteria** [14]. This tool evaluates 10 key domains, including:<br><br>1. Clear and answerable research question<br>2. Comprehensive description of competing alternatives<br>3. Evidence of program effectiveness<br>4. Identification of all relevant costs and consequences<br>5. Accurate measurement of costs and consequences<br>6. Credible valuation of costs and consequences

PRISMA 2020 Checklist

Section and Topic	Item #	Checklist item	Location where item is reported
			7. Adjustment for differential timing of costs and consequences<br>8. Incremental analysis of alternatives<br>9. Sensitivity analysis<br>10. Adequate presentation and discussion of results.<br><br>Each criterion was rated as **Yes (+), No (-), or Cannot tell/N/A**, with studies receiving ≥50% positive ratings classified as **strong methodological rigor**, and those with <50% as **lower methodological quality**.<br><br>**Two reviewers (S.P. and M.U.) independently assessed the quality of each study.** Any disagreements were resolved through discussion or consultation with a third reviewer. No automation tools were used in the risk-of-bias assessment.
Effect measures	12	Specify for each outcome the effect measure(s) (e.g. risk ratio, mean difference) used in the synthesis or presentation of results.	**location in manuscript:** *Results → Length of Stay (LOS) and Cost of Hospitalization; Tables 1 & 2*<br><br>For the outcomes of **hospital length of stay (LOS)** and **hospitalisation costs**, the effect measures reported in the included studies were primarily **continuous measures**:

Section and Topic	Item #	Checklist item	Location where item is reported
			• **Length of Stay (LOS):** reported as **mean days**, with ranges or standard deviations provided when available.<br><br>• **Cost of Hospitalisation:** reported as **mean cost per patient** in local currencies (USD, AUD, GBP, EUR), with ranges reported where available.
Synthesis methods	13a	Describe the processes used to decide which studies were eligible for each synthesis (e.g. tabulating the study intervention characteristics and comparing against the planned groups for each synthesis (item #5)).	**Location in manuscript:** *Materials and Methods → Search Strategy; Inclusion/exclusion criteria; Results → Search Outcomes:*<br><br>To decide which studies were eligible for inclusion in each synthesis, all retrieved studies were first screened for relevance to the review question: "What are the costs of hospitalisation and length of hospital stay caused by dental infection?"<br><br>• **Step 1 – Screening Titles and Abstracts:** Two reviewers (S.P. and M.U.) independently screened all titles and abstracts to identify potentially relevant studies.<br><br>• **Step 2 – Full-text Assessment:** Full texts of

PRISMA 2020 Checklist

Section and Topic	Item #	Checklist item	Location where item is reported
			25 potentially eligible articles were retrieved and evaluated against pre-defined inclusion and exclusion criteria, focusing on study population (patients hospitalised due to dental/odontogenic infections), outcomes (length of hospital stay and hospitalisation costs), study type (retrospective or prospective analyses), and publication language (English).<br>• **Step 3 – Tabulating Study Characteristics:** Key characteristics of each study (country, study duration, sample size, patient age and gender, outcomes measured, and cost reporting) were tabulated. Studies were compared against the planned outcome domains for synthesis (LOS and cost) to determine eligibility for inclusion in quantitative or narrative synthesis.<br>• **Step 4 – Independent Review and Consensus:** Any disagreements between reviewers were resolved through discussion. Studies that reported relevant outcomes but did not meet

Section and Topic	Item #	Checklist item	Location where item is reported
			criteria for both LOS and cost outcomes were excluded from the synthesis for that outcome, though still noted for descriptive purposes.<br><br>• **Step 5 – Synthesis Decision:** Only studies that reported LOS or cost data clearly enough to allow tabulation or comparison were included in the synthesis. Studies with missing or unclear outcome data were noted, and no imputation or automated tools were used to infer missing values.
	13b	Describe any methods required to prepare the data for presentation or synthesis, such as handling of missing summary statistics, or data conversions.	**Location in manuscript:** *Materials and Methods → Data Extraction; Results → Length of Stay (LOS) and Cost of Hospitalization*<br><br>Before synthesis, extracted data were prepared as follows:<br><br>• **Currency conversions:** Hospitalisation costs reported in different currencies (AUD, USD, GBP, EUR) were presented as reported in the original studies. No direct currency conversion or inflation

Section and Topic	Item #	Checklist item	Location where item is reported
			adjustment across studies was performed to allow fair comparison of trends rather than absolute cost values.<br><br>• **Handling missing data:** Where outcome data were missing or unclear (e.g., cost not reported, sample age range not fully defined), these were noted as "N/A" in the summary tables. No imputation was performed.<br><br>• **Standardising units and formats:** Length of stay was uniformly presented in days. Costs were presented per patient, consistent with the original study reporting. Where studies reported multiple time points or subgroups, only the overall mean or total for the relevant outcome was extracted.<br><br>• **Data tabulation:** Extracted data (age, gender, LOS, costs, mortality) were organized into structured tables (Tables 1–2) to facilitate narrative synthesis. No automated tools were used; all conversions and tabulations were manually verified by two reviewers.

Section and Topic	Item #	Checklist item	Location where item is reported
	13c	Describe any methods used to tabulate or visually display results of individual studies and syntheses.	**Location in manuscript:** *Materials and Methods → Data Extraction; Results → Length of Stay and Cost of Hospitalization*<br><br>Extracted data from included studies were prepared for presentation and synthesis as follows:<br><br>• **Standardisation of outcomes:** Length of hospital stay (LOS) was uniformly reported in days across all studies. Cost data were presented as reported in the original studies (AUD, USD, GBP, EUR). No direct conversion between currencies or adjustment for inflation was performed, but values were noted alongside the relevant currency and study period for transparency.<br><br>• **Handling missing or unclear data:** Where studies did not report specific outcomes (e.g., cost or LOS), these were recorded as "N/A" in the summary tables. No imputation was performed.<br><br>• **Tabulation for synthesis:** Key variables (age, gender, LOS, costs, mortality) were

PRISMA 2020 Checklist

Section and Topic	Item #	Checklist item	Location where item is reported
			systematically tabulated in Tables 1–2 to facilitate narrative synthesis and comparison.<br><br>• **Verification:** Data extraction and preparation were conducted independently by two reviewers to ensure accuracy. No automated tools were used for data conversion or synthesis.
	13d	Describe any methods used to synthesize results and provide a rationale for the choice(s). If meta-analysis was performed, describe the model(s), method(s) to identify the presence and extent of statistical heterogeneity, and software package(s) used.	**Location in manuscript:** *Materials and Methods → Data Extraction; Results → Length of Stay and Cost of Hospitalization*<br><br>**Answer:**<br><br>Results from included studies were synthesized using a **narrative descriptive approach**. Quantitative meta-analysis was **not performed** due to heterogeneity across studies in terms of study design, sample size, population characteristics, geographic location, outcome measures, currency of reported costs, and time periods.<br><br>• **Rationale:** The variability in study populations (e.g., children vs adults), reporting of costs in different currencies (AUD, USD, GBP, EUR), and differences in healthcare

Section and Topic	Item #	Checklist item	Location where item is reported
			settings precluded pooling of data.<br><br>• **Synthesis approach:** Key variables such as length of stay (LOS), hospitalisation costs, age, gender, and mortality were systematically tabulated (Tables 1–2) and summarized narratively to highlight patterns, ranges, and trends. Comparisons were made where appropriate, with attention to contextual factors such as healthcare system differences.<br><br>• **Software:** Endnote was used for managing references and duplicates, and Microsoft Excel was used to organize and tabulate extracted data for synthesis.
	13e	Describe any methods used to explore possible causes of heterogeneity among study results (e.g. subgroup analysis, meta-regression).	**Location in manuscript:** *Results → Length of Stay and Cost of Hospitalization; Discussion → Factors Affecting LOS and Costs*<br><br>Heterogeneity among study results was explored **qualitatively** due to differences in study design, population characteristics, healthcare settings,

PRISMA 2020 Checklist

Section and Topic	Item #	Checklist item	Location where item is reported
			currencies, and outcome reporting. Subgroup comparisons were conducted narratively based on:<br>• **Geographic location** (e.g., USA, Australia, Europe, Brazil)<br>• **Age group** (children vs adults)<br>• **Severity of infection** (high-risk vs low-risk, ICU admission)<br>• **Healthcare setting characteristics** (hospital protocols, surgical vs non-surgical management)<br>The impact of these factors on outcomes such as **length of stay (LOS)** and **hospitalisation costs** was summarized descriptively. Meta-regression or formal statistical subgroup analyses were **not performed** due to the retrospective design and high variability in the reported data.
	13f	Describe any sensitivity analyses conducted to assess robustness of the synthesized results.	**Location in manuscript:** *Methods → Quality Evaluation; Results → Length of Stay and Cost of Hospitalization*

PRISMA 2020 Checklist

Section and Topic	Item #	Checklist item	Location where item is reported
			**Answer:**<br><br>No formal statistical sensitivity analyses were conducted due to the retrospective design and the heterogeneity of study populations, outcome measures, and reporting formats. However, **robustness of the synthesized results** was assessed qualitatively by comparing outcomes across:<br><br>• **Geographic regions** (USA, Australia, Europe, Brazil)<br>• **Age groups** (children vs adults)<br>• **Severity of infection** (high-risk vs low-risk, ICU admission)<br>• **Healthcare system characteristics** (hospital protocols, surgical vs non-surgical management) The narrative synthesis highlighted consistent trends in length of stay and hospitalization costs across these subgroups, supporting the reliability of the main conclusions despite variability in individual study designs.

Section and Topic	Item #	Checklist item	Location where item is reported
Reporting bias assessment	14	Describe any methods used to assess risk of bias due to missing results in a synthesis (arising from reporting biases).	**Location in manuscript:** *Methods → Quality Evaluation; Results → Search Outcomes and Tables 1 & 2*<br><br>**Answer:**<br><br>Risk of bias due to missing results (reporting bias) was considered by examining the completeness of reported outcomes across studies. Specifically:<br><br>• All eligible studies were screened for **length of stay** and **hospitalization costs**; studies not reporting these outcomes were excluded from synthesis.<br><br>• Multiple sources (Web of Science, Medline via Ovid, Google Scholar) were searched to minimise publication bias.<br><br>• Comparison of reported vs expected outcomes (e.g., total hospital charges, ICU admissions) helped identify potential selective reporting.<br><br>• No formal statistical methods (e.g., funnel plots) were applied due to the small number of studies and heterogeneity of outcome measures.

Section and Topic	Item #	Checklist item	Location where item is reported
			Overall, reporting bias was assessed qualitatively, acknowledging that missing or inconsistently reported cost data across studies could influence the synthesized estimates.
Certainty assessment	15	Describe any methods used to assess certainty (or confidence) in the body of evidence for an outcome.	**Location in manuscript:** *Methods → Quality Evaluation; Results → Summary of Included Studies and Tables 1 & 2*<br><br>**Answer:**<br><br>Certainty (confidence) in the body of evidence for each outcome—hospital length of stay and hospitalization costs—was assessed using a modified version of the **Drummond and Jefferson economic evaluation criteria**. Each study was rated across 10 methodological quality domains (e.g., clarity of research question, comprehensiveness of cost identification, accuracy of cost measurement). Studies achieving ≥50% positive ratings were considered to have **strong methodological rigor**, while those below this threshold were deemed lower quality. Certainty assessments were qualitative, taking into account study design (all retrospective), sample size, completeness of outcome reporting, and consistency across studies. No formal GRADE or statistical certainty scoring was applied; instead,

Section and Topic	Item #	Checklist item	Location where item is reported
			judgments were based on methodological robustness and the risk of bias in reported outcomes.
**RESULTS**			
Study selection	16a	Describe the results of the search and selection process, from the number of records identified in the search to the number of studies included in the review, ideally using a flow diagram.	**Location in manuscript:** *Results → Search Outcomes; Figure 1 (PRISMA Flow Diagram)*<br><br>**Answer:**<br><br>The initial database search across **Web of Science, Medline via Ovid, and Google Scholar** yielded **125 records**. After **removing duplicates**, titles and abstracts were screened by two independent reviewers (S.P. and M.U.), resulting in **25 full-text articles** assessed for eligibility. Following full-text review, **16 retrospective studies** met the inclusion criteria and were included in the systematic review. The included studies spanned multiple countries, including the **USA (6), Australia (4), Brazil (1), Germany (1), Poland (1), Lithuania (1), Turkey (1), and the UK (1)**, accounting for a cumulative total of **156,487 hospitalizations** due to dental infections.<br><br>A **PRISMA flow diagram (Figure 1)** visually illustrates the search and study selection process, detailing the numbers of records identified, screened, excluded, and included.

Section and Topic	Item #	Checklist item	Location where item is reported
	16b	Cite studies that might appear to meet the inclusion criteria, but which were excluded, and explain why they were excluded.	**Location in manuscript:** *Materials and Methods → Inclusion/exclusion criteria*<br><br>**Answer:**<br><br>During full-text screening, several studies appeared to meet the inclusion criteria but were subsequently excluded for the following reasons:<br><br>1. **Seppänen et al., 2011 [2]** – Focused on the risk of infection spread from dental treatment rather than hospitalisation costs or length of stay.<br><br>2. **Obayashi et al., 2004 [7]** – Examined the spread of odontogenic infections via imaging but did not report hospitalisation length or cost outcomes.<br><br>3. **Acharya & Khan, 2015 [8]** – Case reports and narrative review; lacked quantitative data on hospitalisation or costs.<br><br>4. **Pucci et al., 2021 [32]** – Focused on pregnancy-related outcomes and maternal/fetal mortality; costs and hospital LOS were not reported.

PRISMA

PRISMA 2020 Checklist

Section and Topic	Item #	Checklist item	Location where item is reported
Study characteristics	17	Cite each included study and present its characteristics.	**Location in manuscript:** *Results → Table 1*
Risk of bias in studies	18	Present assessments of risk of bias for each included study.	**Location in manuscript:** *Materials and Methods → Quality Evaluation; Results → Table 3 (Risk of Bias)*<br><br>**Answer:**<br><br>The risk of bias in the 16 included retrospective studies was assessed using a **modified Drummond & Jefferson checklist** for economic evaluations. Each study was independently assessed by two reviewers (S.P. and M.U.) using 10 criteria covering the clarity of the research question, completeness of cost and outcome measurement, credibility of valuation, timing adjustments, incremental analysis, sensitivity analysis, and reporting transparency. Any discrepancies between reviewers were resolved through discussion. Studies with ≥50% positive ratings were classified as **low risk of bias**, and those with <50% positive ratings were classified as **high risk of bias**.<br><br>**Author (Year)** / **Risk of Bias Assessment (Modified Drummond & Jefferson)** / **Overall Quality Rating**<br>Ahmad et al., 2013 [15] / + + + + + - + N/A - + / Moderate

PRISMA 2020 Checklist

Section and Topic	Item #	Checklist item	Location where item is reported		
			Christensen et al., 2013 [16]	+ + + + + + + + + +	Low
			Shah et al., 2013 [17]	+ + + + + - + + + +	Low
			Gonçalves et al., 2013 [18]	+ + N/A + - - + N/A - +	High
			Kara et al., 2014 [19]	+ + + + - - + N/A - +	Moderate
			Kruger & Tennant, 2015 [20]	+ + + + + + + + + +	Low
			Rūta Rastenienė et al., 2015 [21]	+ + + + + - + + N/A +	Moderate
			Gams et al., 2017 [22]	+ + + + + + + + + +	Low
			Liau et al., 2018 [23]	+ + + + + - + + + +	Low
			Doll et al., 2018 [24]	+ + + N/A + - + + + +	Moderate
			Nadig & Taylor, 2018 [25]	+ + + + + - + N/A - +	Moderate
			Morón et al., 2019 [26]	+ + + + + + + + + +	Low
			Han et al., 2019 [12]	+ + + + + + + + + +	Low
			Fu et al., 2020 [27]	+ + + + + - + + + +	Low

Section and Topic	Item #	Checklist item	Location where item is reported
			Zawiślak & Nowak, 2021 [28] + + + + - - + N/A - + Moderate<br>Neal et al., 2022 [29] + + + + + + + + + + Low<br>**Legend:**<br>• + = Criterion met (low risk of bias)<br>• - = Criterion not met (high risk of bias)<br>• N/A = Not applicable or unclear<br>• Overall quality rating:<br>○ **Low risk of bias** = ≥50% positive criteria<br>○ **Moderate risk of bias** = 50% positive criteria<br>○ **High risk of bias** = <50% positive criteria<br>The **average rating** across all studies was **6.5/10**, indicating that most included studies exhibited **moderate to low risk of bias**. Common limitations included incomplete reporting of

Section and Topic	Item #	Checklist item	Location where item is reported
			sensitivity analyses and inconsistent valuation of costs across studies
Results of individual studies	19	For all outcomes, present, for each study: (a) summary statistics for each group (where appropriate) and (b) an effect estimate and its precision (e.g. confidence/credible interval), ideally using structured tables or plots.	Table 1
Results of syntheses	20a	For each synthesis, briefly summarise the characteristics and risk of bias among contributing studies.	“Materials and Methods (Quality Evaluation), Results (Search Outcomes, LOS and Cost of Hospitalization), Tables 1 and 2.” **Summary of Characteristics and Risk of Bias for Contributing Studies:**<br><br>The review included **16 retrospective studies** published between 2013 and 2022, covering a total of **156,487 hospitalizations** due to dental infections. Geographically, six studies were from the **USA**, four from **Australia**, and the remaining from **Brazil, Germany, Poland, Lithuania, Turkey, and the UK**. Sample sizes varied widely, ranging from **20 to 65,000 patients**, with mean ages across studies from **6.3 to 43 years**, reflecting inclusion of both pediatric and adult populations. Gender distributions were generally balanced, although a slight male or female predilection was noted in some studies.<br><br>Hospitalization outcomes included **length of stay (LOS)**, ranging from **1.15 to 10 days**, and **hospitalization costs**, ranging from **AUD $2,402 to USD $47,835.60 per patient**, reported in various currencies. Differences in LOS and costs were influenced by

Section and Topic	Item #	Checklist item	Location where item is reported
			infection severity, age, comorbidities, timing of treatment, and healthcare system characteristics.<br><br>**Risk of bias** was assessed using a modified version of the **Drummond and Jefferson criteria**. Studies were rated as having **strong methodological rigor** if they met ≥50% of criteria, and **lower quality** if they met <50%. The **average quality score** across the 16 studies was **6.5/10**, indicating generally moderate quality. Most studies provided clear research questions, described patient populations and interventions, and measured relevant costs accurately, but limitations included retrospective design, variability in reporting, and incomplete information on certain cost components.<br><br>Overall, the contributing studies were **heterogeneous in design, population, and outcomes**, but collectively provided sufficient data to synthesize the **economic and hospitalization burden of dental infections**.
	20b	Present results of all statistical syntheses conducted. If meta-analysis was done, present for each the summary estimate and its precision (e.g. confidence/credible interval) and measures of statistical heterogeneity. If comparing groups, describe the direction of the effect.	**Results of Statistical Syntheses:**<br><br>No meta-analysis was performed due to the **heterogeneity of study designs, populations, outcome measures, and currencies reported**. Instead, a **narrative synthesis** was conducted, summarizing **length of hospital stay**

PRISMA 2020 Checklist

Section and Topic	Item #	Checklist item	Location where item is reported
			**(LOS)** and **hospitalization costs** across the included studies.
	20c	Present results of all investigations of possible causes of heterogeneity among study results.	**Discussion section; • Paragraphs discussing variability in LOS and costs**:<br><br>"Multiple factors influence hospital Length of Stay (LOS), including age, comorbidities, infection severity, and timing of intervention. Han et al. [12] noted that 64.1% of hospitalised patients were high-risk due to airway-related space involvement. Most underwent surgical drainage and tooth extraction under general anaesthesia (74.5%). Kara et al. [19] demonstrated that extractions performed within 48 hours significantly reduced LOS."<br><br>• **Paragraphs discussing demographic and clinical factors affecting outcomes**:<br><br>"Older patients tend to have longer stays, often due to large-space abscesses and systemic comorbidities [12]. Additionally, conditions like diabetes, obesity, and immunosuppression have been associated with increased LOS and hospital costs [41]. Higher odontogenic infection severity scores and ASA scores (≥3) also correlated with increased healthcare expenses."

Section and Topic	Item #	Checklist item	Location where item is reported
			• **Paragraphs discussing gender and health-seeking behavior differences**: "Gender differences emerged in health-seeking behaviours. A review by Lipsky [42] found that men are more likely to neglect oral health, delay seeking care, and present with more severe conditions, leading to higher hospitalisation rates in 11 of 16 studies [43–47]. However, some studies with larger sample sizes observed slightly higher hospitalisation rates among women [17,20,26]."
	20d	Present results of all sensitivity analyses conducted to assess the robustness of the synthesized results.	Not applicable /
Reporting biases	21	Present assessments of risk of bias due to missing results (arising from reporting biases) for each synthesis assessed.	**Risk of bias due to missing results:** "No formal assessment of risk of bias due to missing results or reporting biases was performed. Some included studies did not report all outcomes of interest (e.g., cost per patient or length of stay), which is noted in Table 2 and the Results section. This limitation is acknowledged in the discussion
Certainty of evidence	22	Present assessments of certainty (or confidence) in the body of evidence for each outcome assessed.	"The certainty or confidence in the body of evidence was not formally assessed using tools such as GRADE. However, the methodological quality of included studies was evaluated using a modified Drummond and Jefferson checklist, and findings are

Section and Topic	Item #	Checklist item	Location where item is reported
			summarised in the Results section (Quality Evaluation). Studies with ≥50% positive ratings were considered to have strong methodological rigour, while those with <50% positive ratings were deemed lower quality.”
**DISCUSSION**			
Discussion	23a	Provide a general interpretation of the results in the context of other evidence.	**Discussion section**, especially the paragraphs starting with:<br>• “Despite advancements in dental and medical care and the widespread use of antibiotics, the reviewed literature highlights a rising trend in the incidence, severity, and cost of odontogenic infections requiring hospitalisation [5].”<br>• “Overall, this review reveals the multifactorial burden of odontogenic infections on healthcare systems and underscores the importance of timely, source-focused treatment.”<br>• “In conclusion, Dental infections impose a significant burden on healthcare systems, with hospital length of stay ranging from 1.15 to 10 days and costs varying from

PRISMA 2020 Checklist

Section and Topic	Item #	Checklist item	Location where item is reported
			AUD 2,402 to USD 47,835.60.
	23b	Discuss any limitations of the evidence included in the review.	**Discussion section**, particularly where noted:<br>• "The reviewed studies revealed substantial variability in these outcomes, influenced by infection severity, healthcare infrastructure, and geographic factors."<br>• "Some studies included only children or specific age groups, affecting generalisability."<br>• "The cost of hospitalization was reported in pounds, Australian dollars, Euros, and US dollars. However, these costs may not be directly comparable to current values due to the time gap between the data collection and publication dates, as well as variations in currency values across countries."<br>• References to retrospective study designs and limitations in reporting are also throughout the **Discussion** and **Results**

Section and Topic	Item #	Checklist item	Location where item is reported
			sections where study characteristics are summarised (Tables 1 and 2).
	23c	Discuss any limitations of the review processes used.	**limitations of the review processes used** are implicitly reported in the **Materials and Methods** and **Discussion** sections. Specifically:<br>• **Materials and Methods / PRISMA Compliance:**<br>○ "The review protocol was not prospectively registered." → This is a limitation because registration reduces risk of bias in review processes.<br>• **Discussion section:**<br>○ "The reviewed studies revealed substantial variability in these outcomes, influenced by infection severity, healthcare infrastructure,

PRISMA 2020 Checklist

Section and Topic	Item #	Checklist item	Location where item is reported
			and geographic factors.” → Highlights limitations in comparability due to heterogeneity.<br>○ “Some studies included only children or specific age groups, affecting generalisability.” → Indicates a limitation in inclusion criteria affecting review conclusions.<br>○ Retrospective nature of all included studies is noted in **Results** → Limits the strength of evidence and review conclusions.
	23d	Discuss implications of the results for practice, policy, and future research.	**Location in manuscript:**<br>• **Discussion:** Paragraphs discussing timeliness of care, risk-stratification,

Section and Topic	Item #	Checklist item	Location where item is reported
			preventive strategies, and socioeconomic impact.<br><br>• **Conclusion:** Paragraph summarising the burden of disease, variability in costs and LOS, and the need for standardised tools and policy awareness.
**OTHER INFORMATION**			
Registration and protocol	24a	Provide registration information for the review, including register name and registration number, or state that the review was not registered.	**Methods / PRISMA Compliance** section:<br><br>• **Statement:** "The review protocol was not prospectively registered."<br><br>**Location in manuscript:**<br><br>• **Materials and Methods → PRISMA Compliance** section, 2nd paragraph.
	24b	Indicate where the review protocol can be accessed, or state that a protocol was not prepared.	• **Reported in manuscript:** Materials and Methods → PRISMA Compliance.<br><br>• **Content:** "The review protocol was not prospectively registered."<br><br>• **Checklist note:** No protocol was prepared.

PRISMA 2020 Checklist

Section and Topic	Item #	Checklist item	Location where item is reported
	24c	Describe and explain any amendments to information provided at registration or in the protocol.	• **Reported in manuscript:** Not applicable.<br>• **Checklist note:** No protocol existed; therefore, no amendments were made.
Support	25	Describe sources of financial or non-financial support for the review, and the role of the funders or sponsors in the review.	Not reported in manuscript. "No financial or non-financial support was received."
Competing interests	26	Declare any competing interests of review authors.	• Not reported in manuscript.<br>"The authors declare no competing interests."
Availability of data, code and other materials	27	Report which of the following are publicly available and where they can be found: template data collection forms; data extracted from included studies; data used for all analyses; analytic code; any other materials used in the review.	Not reported :"Data collection forms, extracted data, and analytic materials are not publicly available."

From: Page MJ, McKenzie JE, Bossuyt PM, Boutron I, Hoffmann TC, Mulrow CD, et al. The PRISMA 2020 statement: an updated guideline for reporting systematic reviews. BMJ 2021;372:n71. doi: 10.1136/bmj.n71. This work is licensed under CC BY 4.0. To view a copy of this license, visit https://creativecommons.org/licenses/by/4.0/
